# Motivational Interviewing for Adherence Problems in Cystic Fibrosis; Evaluation of Training Healthcare Professionals

**DOI:** 10.4021/jocmr1603w

**Published:** 2013-10-12

**Authors:** Alistair James Aitken Duff, Gary Joseph Latchford

**Affiliations:** a Leeds Teaching Hospitals NHS Trust, United Kingdom

**Keywords:** Motivational interviewing, Training, Adherence, Cystic fibrosis

## Abstract

**Background:**

Motivational interviewing (MI) offers effective strategies for enhancing behaviour change and is particularly useful for patients who exhibit poor adherence. This study evaluated MI training for cystic fibrosis (CF) teams, which comprised of one 4-hour workshop on MI principles, followed 6 months later by another on applying MI during brief consultations.

**Methods:**

Health professionals (N = 60) from 7 teams completed questionnaires on learning outcomes 6 months after the first workshop, but before the second. Eleven participated in telephone-interview, 3 months after the second workshop.

**Results:**

Quantitative analysis showed all participants used MI with a patient at least once after the first workshop and felt the approach was potentially helpful. Although self-appraisal of skill and confidence in MI was ‘moderate’, all felt confident in their ability to develop their skills and almost all intended to use MI in the future. Qualitative analysis confirmed the positive experiences of training and of using MI in practice, particularly in relationship building. However MI was utilised depending on team support and workload pressures.

**Conclusions:**

This study showed that initial MI training with CF team-members resulted in increased knowledge and confidence about acquiring and applying MI techniques. However, this was balanced with consideration of barriers to application, further training needs and ongoing team-based support.

## Introduction

Adherence is a major problem in healthcare and generally thought to be around 50% across all illness groups [1]. Cystic fibrosis (CF) is one condition where adherence is known to be problematic. Healthcare professionals (HCP) in CF require excellent communication and active listening skills, yet receive little explicit training.

CF is an autosomal recessive disorder most common in Caucasian populations. It is caused by mutations in the CF transmembrane conductance regulator (CFTR), resulting in multiple complications but especially in the lungs, where there is impaired mucociliary clearance, chronic pulmonary infection and eventual respiratory failure. Unlike previous decades of care, prognosis has been improving. The UK median survival age is 41.5 years [2] and parents of infants born since 2000 have been told that they can expect their child to live into their mid-50s [3]. However, achieving these outcomes is contingent upon optimal adherence to a time-consuming treatment regime which includes daily enzyme replacement therapy, high-fat intake requirements, airway clearance therapy and the use of several medications to maintain lung health (for example, aerosolised bronchodilators, antibiotics and mucolytics) [4]. Adherence to nebulised therapies is particularly low [5-7] and proliferation of new medicines and other pharmacological agents currently in development will further add to the burden of treatment for people with CF. Consequently, healthcare professionals in multidisciplinary CF teams (for example, physicians, nurses, physiotherapists and dietitians), who often have long-lasting relationships with patients, must work with them and their families to optimise a clinically effective regimen.

Motivational interviewing (MI) is a framework for facilitating behaviour change in patients who are ambivalent or resistant [8]. Using active listening strategies, healthcare professionals practicing MI help patients to explore discrepancies between beliefs and behaviours, and resolve them by moving towards change. Throughout, healthcare professionals use a non-confrontational style. Unlike purely patient-centred approaches, however, MI enables the use of direct, problem-solving skills in actively supporting patients who express readiness to change.

MI has been successfully utilised in a variety of illness populations such as diabetes [9] and HIV [10] and has reported efficacy and utilisation in CF groups [11, 12]. It is currently undergoing randomised controlled trial due to complete in 2016 [13]. As such, MI has become increasingly familiar to many healthcare professionals. Despite this familiarity, however, MI is not routinely used in healthcare communication [14] and reports of attempts to introduce it have shown mixed results. Good long-term utilisation has been reported amongst general practitioners where half-day follow-up sessions were organised in the following year [15]. However studies of single-event training for nurses have showed that whilst professionals found MI enriching, it was demanding and difficult to maintain the techniques of expressing empathy over simple advice-giving [16]. Another evaluation of 117 video-taped nurse-patient consultations showed only partial use of MI techniques with substantial variation being observed between constructs [17].

What is clear is that if MI is to be practiced widely in CF care, training and support needs to be in place to further increase willingness to adopt it [7]. The purpose of this article is to describe the implementation of MI training for healthcare professionals (HCPs) working in CF teams across the UK and the evaluation of longer-term learning outcomes and clinical practice.

## Materials and Methods

### Programme development

In recognition of the necessity to foster holistic patient-HCP relationships, the MI training programme placed emphasis on the need to understand the factors associated with sub-optimal adherence and how beliefs and perceptions about treatment influence health care behaviours. The programme cited the Necessity-Concern Framework [18-20] which argues that adherence behaviour is influenced by specific beliefs about the ‘need’ for treatment and the ‘concerns’ that the treatment may engender.

If a patient is seen as resistant or un-motivated to change, attempts by their HCP to offer pragmatic solutions can be unproductive, leaving the patient feeling poorly understood and the HCP frustrated. Consequently the training programme incorporated preliminary material on underpinning the value of active patient-engagement and collaboration in understanding the reasons behind poor adherence and agreeing the goals of treatment before any practical efforts were implemented. This was based on establishing both perceptual (for example, beliefs) and practical (for example, lack of time or forgetting) barriers to optimal adherence.

### Programme description

Workshop-formats for MI training have been recommended in a major systematic review of the evidence [21]. The characteristics purported to be effective [21] were assimilated into the current workshops, which included; practice sessions and role-plays, use of a variety of techniques such as videos and giving trainees the opportunity to apply techniques to their own behaviour and reflect on their own attempts to change. A final recommendation [21] is to facilitate opportunities for ongoing feedback and supervision, and whilst this was outside the scope of the workshop facilitators to provide, it was outlined and recommended to participants. Furthermore a two-step workshop approach to MI training has gained good ground in terms of practical delivery to shift-based clinicians and content development [22, 23].

The current training package comprised two four-hour interactive workshops, taking place 6 months apart. It was delivered by two Consultant Clinical Psychologists (AD and GL) who work clinically in paediatric and adult CF centres respectively. GL is an accredited MI Trainer and AD an Advanced MI Practitioner. Sessions were delivered to 73 HCPs, naive to MI, working across 7 CF centres in the UK over an 18 month period (14 sessions in total). The smallest group consisted of 5 participants (Birmingham), the largest, 20 (Bristol); 69 attendees (57 females) provided their contact details at the end of Workshop 1 and agreed to take part in the evaluation. All participants gave consent to participate in the evaluation programme. Ethical approval was not required.

Workshop 1 introduced the four principles of MI: 1), expressing empathy; 2), developing discrepancies (between thoughts and behaviours); 3), dealing (“rolling”) with resistance and; 4), supporting self-efficacy (with a review of key pragmatic solutions to utilise once a patient was ready and willing to change). Each involved opportunities to role-play key techniques. Participants received a purpose written handbook and were invited to practice the MI techniques and feed-back at the next workshop. Workshop 2 had three aims: 1), to review experiences of putting MI into practice; 2) provide refresher material; and 3), consider facilitators and barriers to implementing MI in routine, out-patient practice. A second handbook was written which contained the course material.

### Evaluation

Quantitative: a purpose-designed questionnaire was given to workshop participants at the beginning of the second workshop (namely, 6 months after workshop one). This consisted of 10 items using Likert-scaling to assess their knowledge, beliefs and experience of using MI. Items evaluated: frequency MI had been used, how helpful MI was, importance of using MI with appropriate patients, self-rating of confidence in using MI, understanding of factors that affect adherence, belief that MI can improve adherence, belief in own skills in MI, confidence in ability to improve adherence with patients, belief in ability to develop skills, intention to use MI in the future). Participants returned these questionnaires anonymously.

Qualitative: three months after the second workshop (nine months after workshop one), 11 participants who had completed the questionnaire survey were randomly selected to take part in a semi-structured telephone interview. The interview schedule comprised nine open-ended questions about participants’ experiences of the training workshops (aspects that were ‘helpful’, ‘unhelpful’ or which ‘could have been improved’) of using MI in their own clinical practice (success and failure), their perceptions of barriers and facilitators to using MI in practice, their thoughts about using MI in the future, and any other open-ended reflections. Interviews lasted for an average of 30 minutes and were audio-taped and transcribed. Material was then analysed using thematic analysis [24], a widely used technique for identifying and organising themes in qualitative data.

## Results

### Questionnaire

Sixty participants returned completed questionnaires. All reported using MI with a patient at least once since the first workshop, but the extent to which participants were using it routinely varied, with 16 (27%) using it often, 11 (19%) rarely using it and most (54%) using it occasionally. Only two reported that MI wasn’t helpful, and almost all (55, 92%) felt it important to use MI with patients with adherence problems. Most (44, 73%) indicated they felt only moderately confident in their skills in MI, though this self assessment was made before participation in the second workshop.

Participants agreed that they had increased understanding about adherence (51, 89%) and most felt that MI may be helpful (53, 93%), but self-appraisal of MI skills was mixed with 30 feeling they had the skills to practice MI and others disagreeing or feeling unsure. Most (37, 65%) agreed that they felt more confident that they could help patients with adherence problems, and 100% of respondents felt that they had the ability to develop their MI skills in the future. Intention to use MI in the future was also high with 50 (83%) indicating that they planned to do so.

### Qualitative interviews

Eleven participants from five centres were randomly selected to take part in telephone interviews (8 female; average years in practice 7.2 years). The professional background of participants reflected the professions involved in CF care (Table 1).

**Table 1:** Professional Background and Experience of Interview Participants

	Sex	Experience of CF work (yrs)	Profession
1	F	15	Physician
2	F	2.5	Dietician
3	F	12	Physiotherapist
4	F	7	Nurse
5	F	5	Physiotherapist
6	F	8	Nurse
7	F	2	Dietician
8	F	1	Physiotherapist
9	M	19	Physician
10	M	5	Physician
11	M	3	Social worker

All had completed both workshops and gave written consent to be interviewed via e-mail.

**Figure 1:**
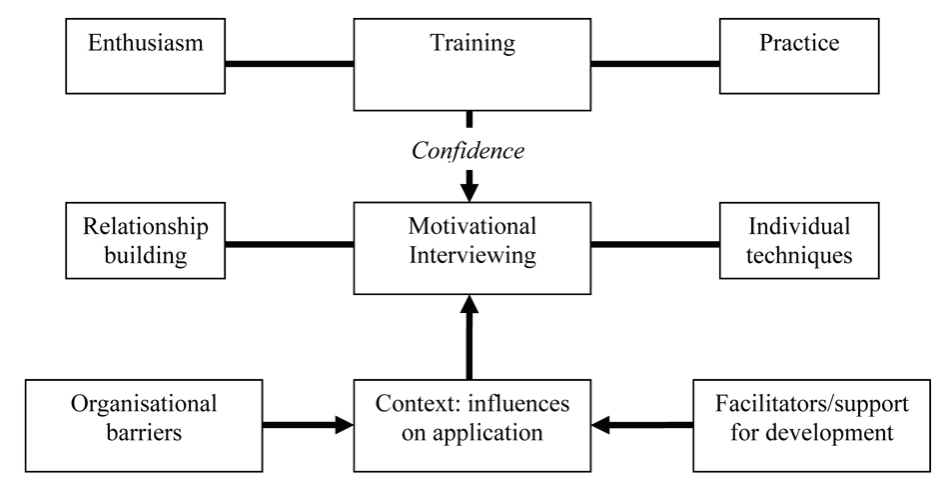
Thematic analysis of telephone interviews.

Analysis of the transcripts of the interviews revealed three main themes (Fig. 1): the practice of MI, the influence of the training on practice, and the context of the workplace.

All participants were positive about using MI. Seven mentioned that they felt it helped them build productive relationships with patients and address adherence issues more effectively. This was commonly cited as a reason for their intention to continue to use MI in their practice. In terms of MI itself, participants cited techniques they particularly valued, including agenda-setting, tools for building rapport, open questions and summarising.

The training was also viewed positively. There was appreciation of opportunities to practice key skills with feedback during both workshops. Participants thought it was particularly helpful that the training materials were written specifically for CF professionals, with examples from clinical practice in CF. Confidence was identified as a key aim in training and this was related to the likelihood that they would continue to use MI routinely. At that time they rated themselves as ‘fairly’ confident in using MI.

Lastly, the context in which participants used MI was also seen as important. Participants valued the opportunity to discuss the use of MI with individual patients with colleagues in their team, and appreciated the way in which training was provided for particular teams rather than mixed groups, in that this encouraged such dialogue. They thought that further training and written or web based resources may facilitate further development, but that the key necessary development would be the adoption of the approach by the team. On the other hand, participants cited a number of potential barriers to using MI. A general feeling was that it was harder to use MI in the clinic setting as opposed to home visits or in-patient work, mainly because of lack of time (to prepare for the session, and to spend with the patient). They noted too that there tends to be a lack of continuity in care, with patients seeing different health professionals at different appointments, making it hard to engage in long term work. Finally, there was also some speculation about whether different professions might find it more difficult to adopt an MI style.

## Discussion

This study is one of only a few to examine the longer-term learning outcomes of MI training for HCPs. It shows that initial training results in increased knowledge and confidence about acquiring and applying MI techniques. However this is balanced with reported barriers to routine application in clinical settings; further training needs and ongoing team-based support. These findings are consistent with previous research which suggested that MI skills are not easily applicable in daily practice without ongoing support. Where this has been put in place, practice has been maintained with good patient outcomes [15]. Four dietitians who participated in the present training who received ongoing supervision from a clinical psychologist maintained their skills leading to lasting change for patients considering nasogastric or gastrostomy feeding and adherence to CF related diabetes care [25].

Attendees were extremely enthusiastic about adopting MI to tackle adherence problems in CF and there was broad agreement that practice-based workshops are a good way of training teams in the techniques which changes practice initially. However, although the majority reported trying to implement MI skills in their practice, it has become clear through the evaluation and discussion in the second workshop that to ensure learning outcomes translate into longer-term changes in clinical practice, consideration needs to be given to providing ongoing support to the team. This might involve training some team members as advanced practitioners in MI, so they may support and supervise the development of colleagues locally.

A potentially more difficult barrier relates to organisational issues such as competing priorities of job plans and staff shortages. Change at a team level is needed if these ideas are to be widely adopted and though some of the changes needed are clearly related to resources, others may be more amenable to more subtle changes in work practices at a team level, such as the allocation of key workers for particular patients to work on adherence issues who would work with them more frequently over an extended period of time.

Another important way forward is to involve the whole team in training and follow-up, and to discuss such barriers at a service level. In this sense, teams can sign up to the ‘spirit’ of MI without necessarily gaining the key skills for day-to-day practice, but which facilitates a context by which those HCP’s who do gain skills feel supported and validated in putting them into routine clinical practice.

The present study is limited by comparatively small numbers of participants and potential for sample bias of those opting into the evaluation process. Measures of competence and/or skills following training were subjective, and more objective evaluation would have strengthened the findings as would an assessment of patient outcomes. However these would have required resources beyond the present scope and offer an opportunity for future research. None-the-less, results add to a growing body of evidence for the potential effectiveness of MI when adopted by HCPs in routine practice and offer clear lessons for practice if MI is to be used in routinely clinical practice. Training needs to encompass the whole team and the context of the service, giving consideration to both ongoing support for clinical skills and ways of prioritising the approach given competing demands on time and resources.
